# The Impact of Sustainable Transformational Leadership on Sustainable Innovation Ambidexterity: Empirical Evidence From Green Building Industries of China

**DOI:** 10.3389/fpubh.2022.814690

**Published:** 2022-02-22

**Authors:** Yuan Tang, Yi-Jun Chen, Yun-Fei Shao, Qiao Cao

**Affiliations:** ^1^School of Management, Sichuan University of Science and Engineering, Zigong, China; ^2^School of Management and Economics, University of Electronic Science and Technology of China, Chengdu, China; ^3^Office of Policy Research, Sichuan Provincial Committee of the Communist Party of China, Chengdu, China

**Keywords:** green building industries, sustainable transformational leadership, sustainable innovation ambidexterity, psychological capital, perceived organizational support

## Abstract

Recognizing that building work will continually encompass, to a certain degree, unfavorable ecological consequences, green building has been encouraged and advocated as a managerial concept to progress in the construction segment. This research created a conceptual model that analyzed whether sustainable transformational leadership (STL) supported sustainable innovation ambidexterity (SIA) in green building industries. This research model was based on organizational support theory, hope theory, social cognitive theory, and attribution theory. This paper aimed to observe the relationship between STL with SIA via the mediating effect of psychological capital (PC). Furthermore, it examined the impact of perceived organizational support (POS) on PC. Moreover, it further examined the relationship between STL and POS. Likewise, it investigated the mediating effect of PC on the relationship between POS and SIA. Finally, it examined POS as a mediator between the relationship of STL and PC. The data for this study were collected from 600 workers employed at green building businesses in China. A questionnaire was delivered to the workers of green building corporations. According to the findings, STL was discovered to have a positive impact on PC. Furthermore, POS had a significant impact on PC. Moreover, PC significantly influenced SIA. Finally, STL was found to be in a significant relationship with POS. The outcomes of this research are extremely beneficial particularly in the situation of developing economies. This research contributes to the existing knowledge that employees with STL exhibit high PC, POS, and SIA in green building industries.

## Introduction

Worldwide resources are utilized at a shocking rate because of humans' overexploitation, and the consequence is not just snowballing greenhouse gas release that is adversely changing the universal climate, but likewise reducing freshwater structures, lessening forest spread, and minimizing fisheries and natural resources (1). To a significant amount, the building business has been accountable for this ecological deterioration (2–5), particularly as a result of its high degree of energy utilization (1). The construction business considerably influences the ecological atmosphere, financial system, and civilization. Worldwide, the building industry utilizes 40% of overall energy production, 12–16% of all the existing water, 32% of unsustainable and sustainable resources, 25% of the overall timber, 40% of the available raw supplies. Furthermore, it generates 30–40% of the entire solid wastes, along with emitting 35–40% of carbon dioxide (6, 7). Recognizing that building work will continually encompass, to a certain degree, unfavorable ecological consequences, green building has been encouraged and advocated as a managerial concept to progress in the construction segment. It is the building segment's reaction to authorizing sustainable growth (7, 8). Green building technologies (SBTs) are an answer to these adverse effects; consequently, keeping in mind some of the previous years, the building business has tried to improve the sustainability of its endeavors through the application of several SBTs (9). According to Ahmad et al. (10), SBTs are the innovative techniques that are integrated into construction strategy to get a sustainable final product, for example, development of construction envelope thermal efficiency, sustainable roof innovations, and solar system. SBTs intend at improving the ecological, societal, and financial functioning of constructions that are three components necessary to focus on the requirement for sustainable advancement in the building business (11, 12). Sustainable construction is the way of designing constructs and utilizing methods that are accountable and efficient in resources through a structure's life-cycle from placement to model, building, management, protection, and restoration (8). During layout and building, sustainable constructions utilize recycled resources, a smaller amount of energy, less water, and efficient resource supply systems. Moreover, it includes integrating water reserving layout and minimizing susceptibility to flooding; minimizing contaminating discharges to the soil, air, and water, and minimizing light and noise contamination (13), thus minimizing the undesirable effect on the ecosystem. Subsequently, this ecological-friendly building method connects economically and socially. Socially, sustainable structures enhance the working and living atmosphere for individuals. Economically, sustainable constructions recommend life cycle expense reductions to holders or occupants (7). Sustainable structures encourage social welfare regarding the flourishing ecosystem and society (14); high-level worker efficiency and minimal absence of employees (15, 16), these advantages are emotive on people, by this it means that transforming sustainable structures are key temptations for project holders (17, 18). Additionally, the green building provides to promotes commercial enterprise since there is a sound connection between return on investment and worker efficiency (19). Consequently, due to the constructive impact of sustainable structures on social characteristics for instance health and efficiency, managers are urged fundamentally to implement SBTs. Social welfare-associated encouragements strengthen the growth of informative green building plans. To promote intellectual development, managers are persuaded to create sustainable learning construction plans to offer ease for employees, pupils, and investigators (7).

There are also certain requirements positioned on green building business contributors to implement sustainable management structure (20) that is currently a fundamental component of corporate policy in various regions and segments. This recent status quo is encouraged by the significant global climate change that causes several risks to social and ecological welfare. Additionally, an environment cognizant management structure has been established as a vital force for the generation of innovative procedures in cost-saving, and revenues growth (21). Though sustainability was primarily perceived as an additional expense to operation, currently corporate managers consider the sustainability approach as a crucial instrument to obtain value. Moreover, earlier studies discover that determining sustainability-associated crisis would proceed to modernization (22). The escalating variability of the peripheral corporate environment is a foremost aspect, which has triggered the recognition of resources and administrative competencies as the principal foundation of sustained competitive gain and the support for corporate policy creation (21). Also, investigators have observed that companies including green building businesses ought to depend on intangible sources to challenge the complication of sustainability matters, implementing a technique to fulfill diverse shareholder pressure (23). Both earlier and recent findings state that business capabilities, psychological characteristics of employees, and the corporate culture perform a substantial role in shaping corporate policy and managerial efficiency (24). The idea of corporations' methodology in management concerns to achieve competitive lead is fairly stated in studies. The increasing consequence of leaders' activities in promoting commitment has been acknowledged by investigators and experts (25, 26). Sustainable transformational leadership (STL) improves worker mindset and the general functioning of green building corporations. Sustainable transformational leaders convey the company's sustainable concept in a smooth outline, explaining the concerns related to strategies required for the attainment of sustainable goals and methodologies regarding the implementation of these strategies. They additionally convey self-confidence and optimism, frequently debating regarding the corporations' sustainable standards, with their workers, and they offer their workers the essential means to accomplish their objectives (27). Sustainable transformational managers deliver adequate prototypes that prepare the employee's psychological situation and encourage the certainty that they can conquer barriers and promote job-appealing activities that attains accomplishment (24).

On the other hand, psychological capital (PC) has recently been stated in the studies of corporate conduct (28). PC can be defined as a knowledge of constructive subjective understanding, optimistic personal characteristics, and encouraging organizations, guarantees to enhance the state of life and avoid the circumstances that occur when life seems to be pointless (29). Positive managerial conduct aims at considering the worker's powers instead of their difficulties (30). Furthermore, via PC's four components comprising of optimism, resilience, hope, and self-efficacy, PC leads to the advancement of individual capital value including individual skills and expertise and social capital targeting the individuals' social system in green building corporations. PC might be handled and capitalized similarly to social and human capital (31). Hope dimension of PC is related to hope theory, which was formed by Snyder and included three elements: aims, methodologies to achieve those aims, and work related to the capabilities to attain aims via those methodologies (32, 33). Hope demonstrated its organizational significance and substantial promotion of PC (34). Moreover, optimism can be linked with Seligman's theory of attribution and can be described as an individual's constructive attribution concerning existing and potential achievements (29). Carver and Scheier (35) described optimism with two important components, namely, pervasiveness and persistence. Peterson (36) indicated that optimism is a practical, adaptable, and dynamical construct and can be built and studied. It is further deeply associated with PC as compared to the other constructs of PC (28). Additionally, it can also enhance workers' efficiency and participation in the organization (37). Likewise, resilience is described by Rutter (38) as individuals' capability to control their situation effectively to safeguard themselves from the adverse outcomes of harmful experiences. Luthans extended this definition and described resilience as the optimistic mental ability to recover from difficulty, dispute, and disappointment or even constructive experiences, development, and enhanced accountability. Furthermore, self-efficacy is developed from Bandura's social cognitive theory. According to this theory, individuals with high-level self-efficacy establish a decent emotional condition of achieving challenging pursuits and having adequate self-confidence to effectively deal with job-associated problems (39). Self-efficacy can be described as a self-belief related to an individual's organized sources, enthusiasm, endeavors and way of activities to achieve any particular job successfully within a certain perspective (40). Previous findings observed that STL affects PC. One explanation might be linked to the contextual standpoint of industries. Hence, it can be inferred that like other service-providing industries, In the green building industrial environment employees are required to offer services at various times. Corporations puts pressure on employees to work well. Hence, workers require managers to offer creativity, assistance, and advice to support them for dealing with these challenges in the organization (41). Transactional leaders, in contrast, are involved with their personal concerns and are not involved in improving their worker's development. PC has been demonstrated in researches to have a better impact as a high-order construct as compared to its parts (42–46). Corresponding to Dollwet and Reichard (42), PC's formation can support companies enhance their employees' interactions (41). Therefore, this study examines the impact of sustainable transformational leadership on the psychological capital of employees.

Perceived organizational support (POS) is described as a worker's awareness of their concern and welfare and the way they are appreciated by their company (47). It is based on the organizational support theory (OST). According to Eisenberger, POS is the worker's faith that their company has an emphasis concerning them that incorporates both interests for welfare, and gratitude and acknowledgment of their roles (48). Equality, supervisory assistance, incentives, and satisfactory work environments were recognized as key originators of POS from the outcomes performed by Rhoades and Eisenberger (49). Equality both procedural and distributive was discovered significantly associated with POS (40). In the areas of human resource and organizational behavior, nevertheless, several types of research independently discover the result of PC, and POS, however, very few researchers have observed the complete and dynamic relations between these constructs in a green building company's environment (40, 50, 51). The earlier investigation results determine that the combination of all four elements of PC delivers an improved calculation for the logical outcomes (52). This study aims to discover the effect of POS on the employees' PC.

Moreover, in the studies of sustainability sustainable innovation is likewise discovered to be of importance in providing sustainable growth owing to the great increase in ecological problems and policies. Sustainable innovation is additionally discovered to be responsible for affecting the corporation's economic, and sustainable efficiency; additionally, it is beneficial in explaining sustainability concerns. Though, merely a few corporations are trained with the broad experience and sources to increase sustainable innovation. Corporations are crucial to strike the appropriate balance between exploitation and exploration to overcome the overdependence on the application of outdated methods correlated to exploitation independently and the ambiguity related to the deployment of exploration methods (53). March (54) was the foremost researcher to modify the concept of “ambidexterity” in the structure of innovation, recommending exploitation and exploration, various experts discovered exploitative and exploratory innovation (55–57). Previous investigations have explored the influence of ambidexterity in corporations with various key topics comprising discovering the most recent information, developing advanced products, supply chain, and increasing the corporation's efficiency (53, 58). This paper aims to explore the concept of ambidexterity in the context of sustainability. Furthermore, sustainable innovation ambidexterity (SIA) has been divided into two components. The methodologies improving or upgrading the corporations' current sustainable resources and knowledge is known as sustainable exploitative innovation. In contrast, the practices employed to attain sustainable innovative knowledge and capabilities are known as sustainable exploratory innovation (56, 57). Consequently, several corporations are exploring sources and knowledge associated with sustainable innovation peripheral to the range of their corporation (55). Centering on the findings of Avey et al. (59) it can be inferred that PC including the psychological traits of the workers can cover the means for the development of SIA in corporations. Moreover, based on earlier studies, a significant association is indicated between the four elements of PC and SIA (60–62). This also verifies the earlier beliefs concerning the point that PC components have collaboration (52, 63). Hence, this research discovers the impact of PC on SIA.

Corresponding to the outcomes of earlier studies, it can be inferred that STL has a positive and significant impact on POS (64, 65). Sustainable transformational leaders increase workers' sense of being encouraged and appreciated by the corporation. If the workers feel a strong association with their leaders, they are prone to be psychologically tied to their companies. Furthermore, it can be stated that STL is helpful as managers have a tendency to believe in employees' skills in handling sustainability issues and respect their roles that paves the way for a higher POS (65). Consequently, STL and POS can be indicated to have a significant relationship (66). This research aims to explore the relationship between STL and POS. Therefore, this research covers research gaps from existing studies. First, this research studies the direct impact of STL and POS on SIA. Second, it investigates the indirect relationship of STL and POS on SIA, via PC used as a mediator. The operational definitions of the research constructs have been indicated in Table 1.

**Table 1:** Operational definitions.

**Construct**	**Definition**	**Source**
Sustainable Transformational Leadership (STL)	STL is an exceptional leadership approach characterized by four qualities incorporating ideal inspiration, logical motivation, enthusiasm, and personalized attention.	(25, 67)
Psychological Capital (PC)	The knowledge of constructive subjective understanding, optimistic personal characteristics, and encouraging organizations guarantees to enhance the state of life and avoid the circumstances that occur when life seems to be pointless	(29)
Sustainable Innovation Ambidexterity (SIA)	sustainable innovation ambidexterity (SIA) has been divided into two components. The methodologies improving or upgrading the corporations' current sustainable resources and knowledge is known as sustainable exploitative innovation. In contrast, the practices employed to attain sustainable innovative knowledge and capabilities are known as sustainable exploratory innovation.	(53, 56, 57)
Perceived Organizational Support (POS)	Perceived organizational support (POS) is described as a worker's awareness of their concern and welfare and the way they are appreciated by their company.	(47)

## Literature Review and Hypothesis Development

### Sustainable Transformational Leadership and Psychological Capital

STL has been extensively recognized as a widespread concept in management studies because of its interpersonal and encouraging style (68). STL is an exceptional leadership approach characterized by four qualities incorporating ideal inspiration, logical motivation, enthusiasm, and personalized attention (25). It indicates that the leaders that possess the strength of motivating personnel to grasp the greatest levels of success and results, reassuring workers to achieve objectives more than their thoughts, supporting workers to deprioritize the self-interest for corporation's shared aims, playing as the organization's motivational force, regarding for innovative dexterity growth between workers and continuously looking for novel prospects for the green building company's advancement (67).

Though the correlation between STL and workers' PC is not investigated by preceding works, the recent studies showed fairly evidently regarding the significant impacts of STL on the combined effect of PC's components. Particularly, regarding self-efficacy, previous findings indicated that STL's personalized contemplation founded on psychoanalysis, supervising, training, and allocation of stimulating duties, helps workers to advance improved self-assurance in their capabilities, particularly in job-explicit competencies to chase aims (52, 61, 69). Furthermore, Le et al. (70) emphasized that transformational leaders employ feelings to persuade their workers to participate in constructive reasoning in times of creating a constructive perspective and recommend innovative concepts. Additionally, by ways of practical guidance and feedback in a considerate and progressive approach of STL personalized involvement, workers tend to produce sensations of optimism (61, 71). Moreover, Le and Lei (71) explained their concerns related to the hope dimension of PC, according to them sustainable transformational managers provide personnel a high level of dependence-built trust and disclosure-built trust in STL, consequently, these leaders can further increase workers' hope by creating together their determination and approach strength to chase envisioned objectives. Likewise, regarding resilience, Bass (25) contended that sustainable transformational managers understand knowledge in innovative approaches, this encourages workers to view barriers as advancing issues. Likewise, workers' assessments of the STL conduct of intelligent motivation give them a wide variety of belief forms and potential explanations to a certain difficulty. This allows them to extremely stick in the direction of their objectives and strongly consider achieving these objectives (61). Various investigations also emphasized the substantial effects of STL on workers' PC (50, 69). Nevertheless, the proof on the STL and PC connection is still inadequate and scarce (72). Hence, in this study, it can be hypothesized that:

**Hypothesis 1**. STL is significantly related to PC.

### Perceived Organizational Support and Psychological Capital

POS and PC concepts have been examined in a few earlier pieces of research. Separately both concepts were discovered to be significantly associated with diverse job-associated results for instance satisfaction, commitment, corporate citizenship conduct, job efficiency, and constructive attitude, and adversely associated with turnover intents, anxiety, worker absenteeism, and stress symptoms. One survey was performed on full-time workers of global traveler hotels in Taiwan, corresponding to the results it was proved that PC was negatively associated with work burnout and PC was in a mediating position on the association between POS and work burnout (73). Furthermore, Liu et al. (74) discovered that PC with its two elements (optimism and resilience) partly mediated POS association for Chinese correctional officials (COs). Moreover, it has also been found that POS and PC of employees have an important role in enhancing job satisfaction (75). Comparable conclusions were described by Yang et al. (76) in their research performed on physical training pupils, corresponding to the outcomes POS and PC both were discovered to decrease sports burnout. Likewise, in one study PC was identified as an antecedent of POS (77). Another study conducted in the information technology professionals context found a significant and positive relationship between POS and PC (78). In additional research, a significant association was found between POS and self-efficacy and the person-organization fit of nurses (40). All of these mentioned studies examined the effect of POS on PC (79), however, the research regarding the green building industrial context is still inadequate and inconsistent. Hence, based on the above-mentioned studies, the following hypothesis can be postulated:

**Hypothesis 2**. POS is significantly related to PC.

### Psychological Capital and Sustainable Innovation Ambidexterity

PC has been demonstrated to have a substantial impact on improving innovation in a corporation (60–62). PC and its elements have been believed to influence workers' innovative conduct. Therefore, innovative behavior seems to be an essential factor for all the organizational levels for fostering innovation; besides, innovation is crucial for the effective and efficient performance of the organization (60). In research on two Pakistani banks, it was discovered that a person with a high level of resilience, self-efficacy, optimism, and hope demonstrates additional innovative and inventive activities in utilizing information technology (IT). In reality, these individuals were further inclined to plan, create, and attain groundbreaking concepts in their IT application procedures (31). Furthermore, in investigations related to PC and innovation, it can be likewise inferred that in green building industries PC has a significant impact on innovation (31, 80). Corresponding to Luthans and Youssef (28), the concept and also organization of innovation varies on a PC transformation in the business. People with constructive PC have a high level of capability to plan and execute advanced concepts for accomplishing the scheduled objectives. These individuals receive the structural variations and can establish novel methodologies for gaining their aims (hope); possess the obligatory self-assurance to practice novel conducts for the attainment of their aims (self-efficacy); advantage from an optimistic sight regarding the potential future (optimism); and familiarize themselves to some novel alteration or effort (resilience). Every one of these qualities is useful in executing the latest concepts in a green building company (31). Though the need for innovation and improvement might encourage anxiety or dissatisfaction amongst workers of a green building organization, a constructive PC as a possibility to take on the hectic challenges, growth, and application of modern concepts appears to be important (63). Hence, the following hypothesis can be postulated.

**Hypothesis 3**. PC is significantly related to SIA.

### Sustainable Transformational Leadership and Perceived Organizational Support

Several investigations have examined the association between transformational leadership and POS (27, 81). Based on the POS theory, it can be inferred that there is a significant association between STL and POS. A leader can be described as a representative of a corporation, and based on the STL conduct of the leader, it can be indicated that the workers have attained constructive treatment from the corporation, which in turn results in a higher POS (47, 64). According to Stinglhamber et al. (65), The sustainable transformational leader guides the workers, takes care of their individual needs, and lets the employees prosper in a compassionate atmosphere. Furthermore, these leaders demonstrate the demand for a greater requirement, prospects, and tasks and inspire workers to innovatively pursue novel possibilities and manage complicated difficulties. Generally, the transformational leader manages to exhibit confidence in workers' capabilities and to evaluate their roles. The environment of encouraging leadership established amongst employees must increase to the entire company, helping to greater POS (66). Consequently, the following can be hypothesized.

**Hypothesis 4**. STL is significantly related to POS.

### The Mediating Role of Psychological Capital Between the Relationship of Sustainable Transformational Leadership and Sustainable Innovation Ambidexterity

Innovation is described as a corporation's capability to create and execute innovative concepts, products, facilities, practices, expertise, managerial systems, strategies, and plans to improve managerial efficiency and attain a sustainable competitive lead (71). Researchers from a broad variety of subjects have tried to understand the fundamental aspects that structure and promote the innovation and improved ability of a corporation (68). Amongst the greatest substantial aspects impacting innovation competencies are identified as STL and PC because they are the strategic and prospective influences for companies to enhance innovation ability and maintain competitive lead in the vigorous corporate world (31, 61, 71). Recent studies likewise demonstrated the indication for the mediation positions of PC in the correlation of leadership with important results of a company for instance innovation expertise, information communicating method, worker creativity, and, organizational citizenship conduct (61). It has also been claimed that companies managed by transformational leaders produce a constructive work environment that is extremely beneficial to promote the constructive psychological and emotive condition of workers for producing innovation and improvement ability (72). Workers' psychological sources are vital fundamental inspirations that empower them to be further innovative and creative at work (52, 80). The previous experimental conclusions have initially established that STL is an essential predictor of workers' PC that further creates better innovation ability for companies. Particularly, amongst components of PC, optimism appears to be a highly efficient mediator in connecting leadership and innovation (69). Still, it can be inferred that the system for the measurement of the way PC mediates the relationship of STL and SIA is not adequate (82). To cover this research gap, the research has recommended a framework to connect STL and SIA through the mediation of PC. Taking into consideration the crucial positions of STL and PC, this research discovers the potential mediation effect of workers' PC between the relationship of STL and SIA in a green building context. Hence, the following hypothesis can be postulated.

**Hypothesis 5**. PC mediates the relationship between STL and SIA.

### The Mediating Role of Psychological Capital Between the Relationship of Perceived Organizational Support and Sustainable Innovation Ambidexterity

POS is the extent to which a corporation considers the welfare of workers, and likewise respects workers' influence and work (47). Additionally, POS is defined as the awareness of workers regarding the benefit, support, and respect experienced by them by the corporation for their efficiency (83). Based on the organizational support theory POS can be described as offering workers reasonable practices, incentives, managerial aid, and encouraging work environments that promote improved emotions of workers being respected and appreciated by the corporation (47). The existing corporate atmosphere is extremely active and workers are confronting unclear work requirements. In this situation, the maintenance and development of social capital is a huge task. To deal with this, corporations are turning out to be extra flexible by shaping strategies adaptable and worker-focused. In these situations of uncertainty, experts must be determined on encouraging psychological forces, which are established and handled efficiently for greater execution (40). Subsequently, in a similar perspective, PC is an essential element of social capital. PC is a possibly optimistic source that contains the skill and power to rapidly respond to an imminent challenge with an optimistic attitude. It has been discovered that PC effects POS (40, 78).

People with great self-efficacy, enthusiasm, and dedication can propose and execute innovative concepts to accomplish their objectives. Furthermore, it is exhibited that self-efficacy is the producer of developing innovative concepts (84). Additionally, several investigations have found the constructive connection of self-efficacy with innovative leaders and companies' development. Jafri (60) likewise discovered a significant association between self-efficacy and workers' innovative conduct. Hopeful individuals tend to face challenges and constantly pursue innovative approaches to achieve their objectives. These individuals generally are searching for innovative concepts to resolve their difficulties. In reality, hopeful workers, regardless of various difficulties in their work, tend to be incredibly passionate regarding innovation (31). Hence, a significant relationship is discovered between hope and innovation also demonstrated a positive relationship between hope and innovative behavior (60). Optimists generate additional optimistic opinions, and expect that great events will occur in the time ahead; they likewise consider that they can manage experiences in their lives. Consequently, these people seldom forget their aspirations in subsequent innovative concepts and search for encouraging and improved ideas in tense situations. Furthermore, optimists demonstrate further strength while confronting difficulties to discover innovative approaches and develop novel prospects (85). Optimism significantly impacts workers' innovation (61). Optimist managers likewise explore innovative concepts to resolve their operational difficulties. Jafri (60) additionally observed a significant correlation between optimism and innovation. Earlier findings proved that resiliency in the work is a great forecaster for work attitudes, efficiency, and additional job results. Individuals with great resilience are capable of adjusting themselves to adjustments, and constantly seek out appreciation and development of innovative concepts due to their greater capability to face uncertainties (31). These individuals primarily are exploring for different events regarding transformation and ambiguity (85); consequently, adaptable workers when confronted with failures, problems, and prospects explore the development of innovative approaches. Resilient managers encourage their employees for uncertainties and innovative conduct (31). Jafri (60) found out a positive relationship between resilience and employees' innovative conduct. Therefore, the following hypothesis can be postulated.

**Hypothesis 6**. PC mediates the relationship between POS and SIA.

### The Mediating Role of Perceived Organizational Support Between the Relationship of Sustainable Transformational Leadership and Psychological Capital

Previously, no studies were found to explore the mediation impact of POS between the relationship of STL and PC. Nevertheless, as workers identify that their company delivers the care and gratitude they want, additionally to the supervisors and managers appreciate their involvement and consideration regarding their welfare. This acknowledgment will generate a feeling of responsibility in workers to be concerned regarding the corporation's prosperity and assist it to accomplish its objectives in a highly innovative manner (66).

Per Rhoades and Eisenberger (49), there are three procedures essential for POS. The first one is related to workers' recognition regarding leaders valuing their influence and welfare, which makes them have a sense of obligation to pay via their conduct. Next, consideration and appreciation assist to fulfill critical specific demands for association and consent at a job. A third viewpoint is that a corporation's identification and endorsement can strengthen workers' confidence that their conduct will be acknowledged and compensated. While transformational leaders accomplish in advocating an atmosphere of POS, workers will be prone to act innovatively to improve the competitive benefit of their corporations (66).

Though, there is a shortage of research on the impact of PC on POS (77, 79). Consequently, there is sufficient capacity for researching analyze the influence of PC on employees' POS in green building industries and various other contexts. Correspondingly, very few investigators examined the association between the three variables including POS, PC, and POS together (51), in which POS had a mediation impact on the association between STL and PC. Consequently, the following can be hypothesized.

**Hypothesis 7**. POS mediates the relationship between STL and PC.

**Figure 1:**
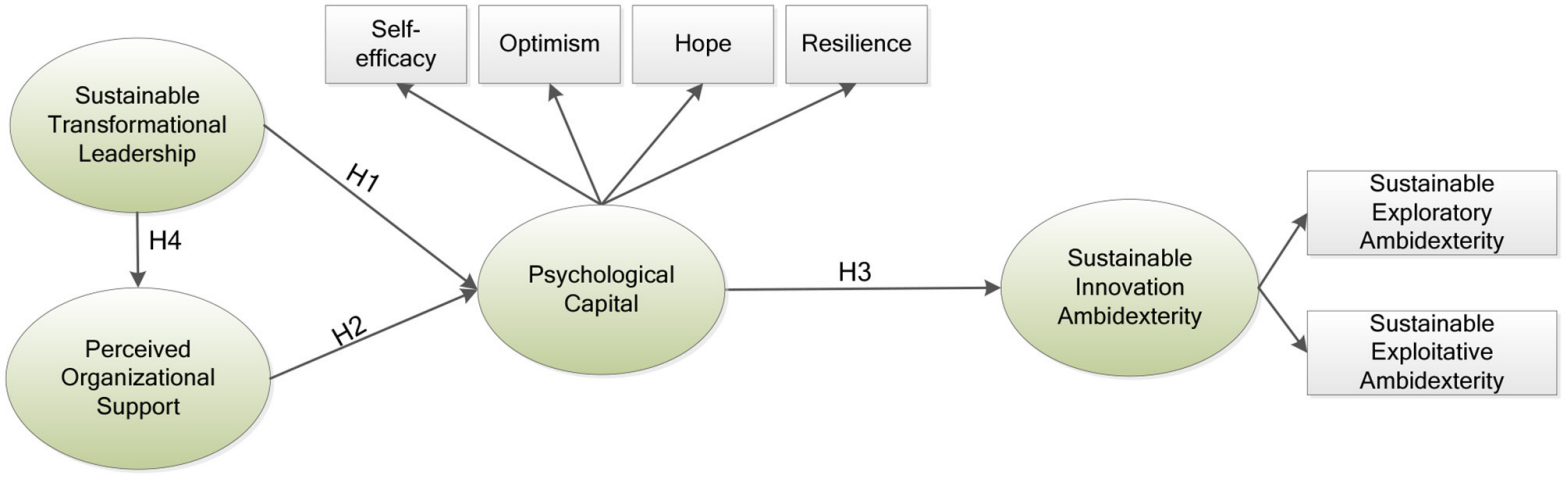
Theoretical framework.

Figure 1 shows the theoretical model for this study.

## Research Methodology

The data for this study was collected from green building businesses located in China. This research concentrates on the workers of various green building corporations in China. A research organization in China was utilized to collect the data for this study. An aggregate of 600 workers was carefully chosen as a sample applying a convenience sampling method. A questionnaire was delivered to the workers of green building corporations. As a replacement for demanding respondents only for their approval to a given measurement item, Likert scale elements questioned, the level of firmness to their approval or opposition, generally on a 7-point gauge from 1 (= strongly disagree) to 7 (= strongly agree), with 4 relating to a neutral group. A pretest was performed via a sample of 120 workers and a response rate of 94.7% was accomplished. STL was measured by the items from Sanjay et al.'s (23) study while the items to assess PC were taken from Luthans et al.'s (86) study. Moreover, POS was measured by the items recommended by Jaiswal et al.'s (87) study. Lastly, items from Khan et al.'s (53) study were utilized to measure SIA.

## Results

### Data Analysis

Two approaches of calculating and estimating partial least squares (PLS) were implemented. In the primary stage, the reliability estimation was conducted. The second phase was related to the descriptive analysis and evaluation of the research model. The aim of the two stages acknowledged previously was to authenticate the construct's reliability, involving confirming the connection among the constructs (88, 89). PLS has been employed and believed as the most excellent means for describing the basic collaboration among variables and thus can simultaneously manage framework variables and measurement items (90). Additionally, as PLS has simpler restrictions for changing normality and ambiguity; it is ideal for investigating the connection if the constructs are irregularly dispersed. Hence, It possesses the benefit of calculating dynamical research frameworks (91). PLS was consequently applicable for this analysis than earlier SEM assessment practices to calculate the relationships between constructs, lessen measurement errors, and avoid collinearity.

### Convergent and Discriminant Validity

The structural equation modeling (SEM) technique is employed to examine the projected hypotheses founded in the aforementioned segment of this research, and as a result, the Smart PLS 3.2.8 was utilized. PLS-SEM procedure is applicable for regular and complex research structures. Similarly, researchers concluded that PLS-SEM is a sustainable practice for measurement as compared to covariance-based SEM. PLS-SEM is regarded fairly in handling and evaluating calculations for mediation effects as compared to regression (2014). PLS-SEM incorporates the inner as well as the outer models. The outer model was calculated by three estimations, including individual item reliability, discriminant validity, and convergent validity. Corresponding to Table 2, the peak value for factor loading is 0.957. The lowest value for factor loading is 0.608. These values are discovered to be above the 0.50 factor loading threshold value (92). Hence, it can be inferred that this research has no concerns regarding individual item reliability. The internal reliability of variables was calculated with the help of composite reliability (CR). The CR value ought to be greater than the threshold value of 0.60 (2014). Corresponding to the CR values of this research indicated in Table 2, the internal consistency of each construct was achieved (93). This study indicates that the internal consistency requirement is fulfilled. Convergent validity suggests the level of similarity between the item and the relative construct. Corresponding to the conclusions revealed in Table 2, 0.567 is discovered as the greatest value of AVE. On the other hand, 0.516 is discovered to be the lowest value of AVE. Subsequently, the AVE values of this research are greater than the threshold value of 0.50 and hence, this study assures convergent validity requirement (2014).

**Table 2:** Construct validity and reliability.

**Constructs**	**Indicators**	**Factor loadings**	**VIF values**	**Composite reliability**	**Average variance extracted (AVE)**
STL	ST1	0.715	1.555	0.877	0.543
	ST2	0.789	1.875		
	ST3	0.728	1.690		
	ST4	0.775	1.765		
	ST5	0.671	1.544		
	ST6	0.737	1.594		
POS	PO1	0.723	1.327	0.809	0.516
	PO2	0.608	1.172		
	PO3	0.817	1.475		
	PO4	0.711	1.333		
SE	SE2	0.731	1.422	0.830	0.549
	SE3	0.744	1.446		
	SE4	0.738	1.524		
	SE5	0.752	1.472		
OP	OP1	0.737	1.404	0.817	0.528
	OP2	0.694	1.539		
	OP3	0.773	1.454		
	OP5	0.701	1.310		
HO	HO3	0.800	1.578	0.834	0.557
	HO4	0.717	1.390		
	HO5	0.691	1.340		
	HO6	0.773	1.497		
RE	RE3	0.769	1.540	0.826	0.543
	RE4	0.713	1.843		
	RE5	0.703	1.349		
	RE6	0.762	1.497		
SEP	SEP1	0.702	1.528	0.872	0.533
	SEP2	0.780	2.001		
	SEP3	0.751	1.870		
	SEP4	0.719	1.552		
	SEP5	0.745	1.745		
	SEP6	0.678	1.717		
SET	SET1	0.708	1.497	0.867	0.567
	SET2	0.833	1.663		
	SET3	0.778	1.431		
	SET4	0.705	1.757		
	SET5	0.734	2.066		
PC	SE	0.832	1.466	0.903	0.700
	OP	0.840	1.426		
	HO	0.826	1.446		
	RE	0.849	1.557		
SIA	SEP	0.957	1.735	0.947	0.899
	SET	0.940	1.682		

*STL, Sustainable transformational leadership; POS, Perceived organizational support; SE, Self-efficacy; OP, Optimism; HO, Hope; RE, Resilience; SEP, Sustainable exploratory innovation; SET, Sustainable exploitative innovation; PC, Psychological capital; SIA, Sustainable innovation ambidexterity*.

This study employed SMART PLS to conduct the analysis. Hence, the common method bias (CMB) was measured with the help of the VIF values. According to the full collinearity assessment approach, a construct's VIF value should be lower than the threshold value of 3.3 to ensure that the framework is free from any CMB (94). Corresponding to the values indicated in Table 2, all the VIF values are lower than 3.3, hence the research model is not affected by CMB (94, 95).

The degree of discrimination among analyzing variables and various construct measures is characterized by discriminatory validity. Table 3 implies a decent discriminant validity for every construct, by indicating that the factor loading value of every item is the highest in the latent structure as compared to other structures (96).

**Table 3:** Cross loadings.

	**STL**	**POS**	**SE**	**OP**	**HO**	**RE**	**SEP**	**SET**
ST1	0.715	0.427	0.333	0.329	0.294	0.416	0.467	0.472
ST2	0.789	0.504	0.381	0.393	0.411	0.499	0.599	0.507
ST3	0.728	0.481	0.345	0.262	0.290	0.370	0.557	0.477
ST4	0.775	0.530	0.396	0.362	0.433	0.437	0.597	0.463
ST5	0.671	0.426	0.282	0.257	0.172	0.292	0.510	0.537
ST6	0.737	0.495	0.397	0.445	0.522	0.428	0.572	0.432
PO1	0.398	0.723	0.395	0.453	0.406	0.452	0.537	0.393
PO2	0.471	0.608	0.203	0.266	0.147	0.277	0.520	0.380
PO3	0.553	0.817	0.461	0.483	0.460	0.477	0.567	0.464
PO4	0.443	0.711	0.295	0.369	0.257	0.265	0.503	0.487
SE2	0.430	0.456	0.731	0.486	0.475	0.475	0.456	0.337
SE3	0.383	0.282	0.744	0.439	0.476	0.435	0.353	0.255
SE4	0.311	0.381	0.738	0.463	0.439	0.458	0.336	0.254
SE5	0.319	0.318	0.752	0.446	0.458	0.487	0.385	0.327
OP1	0.320	0.389	0.403	0.737	0.433	0.370	0.374	0.319
OP2	0.354	0.482	0.477	0.694	0.377	0.455	0.414	0.402
OP3	0.378	0.423	0.464	0.773	0.446	0.465	0.452	0.363
OP5	0.308	0.322	0.452	0.701	0.559	0.479	0.327	0.201
HO3	0.357	0.383	0.524	0.565	0.800	0.510	0.420	0.348
HO4	0.336	0.317	0.443	0.458	0.717	0.448	0.377	0.213
HO5	0.298	0.276	0.446	0.352	0.691	0.477	0.258	0.168
HO6	0.471	0.391	0.446	0.474	0.773	0.524	0.417	0.299
RE3	0.436	0.407	0.511	0.461	0.493	0.769	0.448	0.351
RE4	0.395	0.343	0.429	0.413	0.443	0.713	0.400	0.278
RE5	0.417	0.446	0.457	0.435	0.530	0.703	0.424	0.344
RE6	0.395	0.350	0.446	0.490	0.467	0.762	0.440	0.370
SEP1	0.449	0.557	0.330	0.421	0.271	0.376	0.702	0.584
SEP2	0.550	0.523	0.439	0.464	0.510	0.497	0.780	0.526
SEP3	0.593	0.561	0.350	0.342	0.285	0.458	0.751	0.668
SEP4	0.547	0.572	0.387	0.434	0.380	0.436	0.719	0.576
SEP5	0.625	0.557	0.353	0.246	0.286	0.392	0.745	0.630
SEP6	0.507	0.452	0.408	0.479	0.466	0.383	0.678	0.507
SET1	0.433	0.437	0.285	0.460	0.305	0.303	0.603	0.708
SET2	0.501	0.454	0.293	0.295	0.199	0.313	0.639	0.833
SET3	0.551	0.477	0.324	0.334	0.328	0.366	0.619	0.778
SET4	0.460	0.445	0.385	0.407	0.385	0.411	0.558	0.705
SET5	0.494	0.439	0.209	0.179	0.115	0.333	0.592	0.734

*STL, Sustainable transformational leadership; POS, Perceived organizational support; SE, Self-efficacy; OP, Optimism; HO, Hope; RE, Resilience; SEP, Sustainable exploratory innovation; SET, Sustainable exploitative innovation; PC, Psychological capital; SIA, Sustainable innovation ambidexterity*.

Tenenhaus et al. (97) equation was employed to calculate the goodness of fit (GOF) for this study. The quality of the research framework is analyzed as follows:


(1) $$\begin{array}{r} GOF=\sqrt{\bar{AVE}}\;x\;\sqrt{\bar{R^{2}}}=\;\sqrt{0.542\;x\;0.717}=0.623 \end{array}$$

Subsequent to the aforementioned result, the GOF is 0.623 that attains the 0.376 cut-off requirements for a substantial impact size (98).

### Structural Model Analysis

The path analysis of this research was estimated by employing Smart PLS 3.2.8. The inner model was analyzed and according to researchers, the *p*-value should be lower than the threshold value of 0.05. On the other hand, the *t*-value should be higher than the threshold value of 1.96.

**Figure 2:**
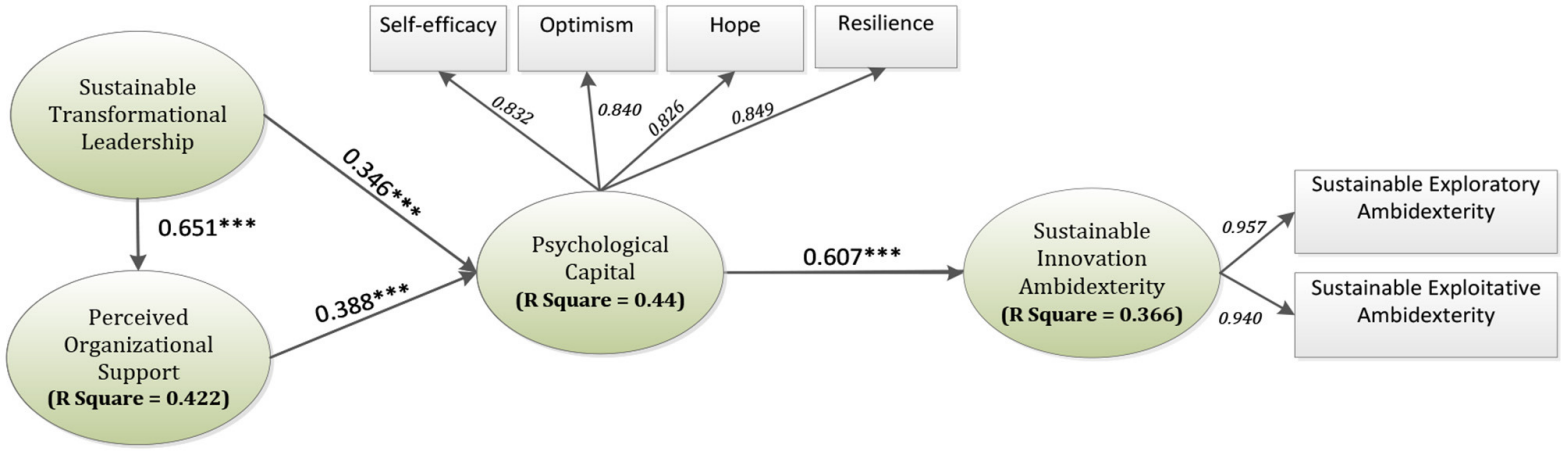
Results of the inner model. ***p-value ≤ 0.001.

As per the results of this research, as shown in Table 4 and Figure 2, STL was discovered to have a positive impact on PC, hence, supporting H1 (β = 0.346, *t*-value = 7.369). Furthermore, POS had a significant impact on PC, therefore H2 (β = 0.388, *t*-value = 8.250) was also supported. Moreover, PC significantly influenced SIA, therefore, H3 (β = 0.607, *t*-value = 16.369) was accepted. STL was found to be in a significant relationship with POS, hence, supporting H4 (β = 0.651, *t*-value = 20.621).

**Table 4:** Hypothesis results.

	**Path coefficients**	***T*-values**	***P*-values**	**Results**
H1: STL -> PC	0.340	7.369	0.000	Supported
H2: POS -> PC	0.391	8.250	0.000	Supported
H3: PC -> SIA	0.606	16.369	0.000	Supported
H4: STL -> POS	0.651	20.621	0.000	Supported

*STL, Sustainable transformational leadership; POS, Perceived organizational support; PC, Psychological capital; SIA, Sustainable innovation ambidexterity*.

The R-square also called the coefficient of determination values for exogenous variables is also highlighted in Figure 2. Corresponding to the results, all the constructs except “Hope” and “SIA” possess values >0.7, which are considered to be a strong effect size. On one hand, “Hope” is considered to have a moderate effect because its value lies between the threshold values of 0.5 and 0.7. On the other hand, “SIA” having a value of 0.366, which lies in between the threshold values of 0.3 and 0.5 indicates a weak effect (99). Furthermore, the factor loadings of all the second-order constructs' indicators are indicated via italic text.

This study utilized Kofod-Petersen and Cassens (100) activity theory to measure the mediation impacts as specified in Table 5. Furthermore, the outcomes of the mediation were also produced by Smart PLS. Corresponding to the outcomes indicated in Table 5, PC fully mediates the relationship between STL and SIA, furthermore, it also mediates the relationship between POS and SIA. Moreover, POS is found to mediate the relationship between STL and PC.

**Table 5:** Indirect effects result.

	**Path**	**Standard**	***T*-values**	***P*-values**
	**coefficients**	**deviation (STDEV)**		
H5: STL -> PC -> SIA	0.206	0.034	6.058	0.000
H6: POS -> PC -> SIA	0.237	0.035	6.699	0.000
H7: STL -> POS -> PC	0.255	0.033	7.740	0.000

*STL, Sustainable transformational leadership; POS, Perceived organizational support; PC, Psychological capital; SIA, Sustainable innovation ambidexterity*.

## Conclusions and Discussions

Numerous behavioral and psychological investigations have been conducted on the associated topic of sustainability. For instance, a study tackled transformational leadership in Rwanda within the context of sustainability. The research was based on the leadership activities of the Leadership Institute (LI), which is a short leadership enhancement course offered to medical practitioners. This LI provided STL concepts to the leaders of Rwanda and assisted them to boost their professional skills (101). Although these studies are not exclusively aiming at green building industries. This implies that a meaningful methodology will be to make use of these concepts as a method of strongly tackling the existing concerns linked to green buildings and related approaches for advancement. This paper aimed to observe the relationship between STL with SIA via the mediating effect of PC. Furthermore, it examined the impact of POS on PC. Moreover, it further examined the relationship between STL and POS. Likewise, it also investigated the mediating effect of PC on the relationship between POS and SIA. Finally, it examined POS as a mediator between the relationship of STL and PC. This research created a conceptual model that analyzed whether STL supported sustainable innovation ambidexterity based on organizational support theory, hope theory, social cognitive theory, and attribution theory. Lastly, the outcomes of this research are extremely beneficial and essential particularly in the situation of developing economies. Certainly, earlier findings suggested that increasing innovation capability for corporations by massive investments in high-tech innovation is not viable because of the majority of corporations in developing nations of small and medium-size and in scarcity of investment and supplies (69, 71). These circumstances cause a higher enthusiasm and challenge to discover cost-effective considerations, which can effectively encourage innovation by corporations in developing economies, in comparison to those in developed economies (71, 102). Workers and their PC are fairly recognized as the major influence that has excellent ability in the innovation development (71, 80, 103). Consequently, adjusting worker mindsets, attitudes, and enthusiasm for innovation by improving their PC by the impact of STL appears to be an ideal and most cost-effective approaches for corporations in developing economies (69).

Corresponding to the results of this study, STL has a significant impact on PC in a green building industrial context. These results are similar to a recent study by Gom et al. (41) conducted in a service industry to discover the effects of transformational leadership on the PC and turnover intents of workers employed in Malaysia. Data were gathered from 162 workers and PLS-SEM analysis to conducted to analyze the associations between the constructs. The conclusions explain that transformational leadership significantly impacts PC and adversely impacts turnover intention.

Additionally, this research likewise implies a substantial association between POS and PC of the green building industries employees. These results are somewhat similar to a study by Priyanka (40), that investigated the mediation effect of POS on the relationship between PC and employee engagement (EE). The data was collected from 420 information technology (IT) service provisioners in India. The gathered data was analyzed using SEM and mediation analysis. The findings of the study substantiated the full mediation of POS on the relationship between PC and EE and revealed that workers with a greater degree of PC, participate more constructively to the POS degree, which additionally improves the worker's degree of commitment at the corporation.

Furthermore, according to the results of this study, PC was in a significant relationship with sustainable innovation ambidexterity. PC demonstrates an evolving development on a constructive methodology to creating and supervising human resources in the job atmosphere of the corporation (104). PC describes the workers' constructive psychological sources described by self-efficacy, optimism, resilience, and hope that is established as the primary inspirations for workers to make and employ innovative and groundbreaking concepts to the company's operating procedures (52, 80). Consequently, developing the PC of people could be an extremely valuable answer and satisfactory option to improve innovation abilities for green building firms (60), particularly in association with developing economies where a greater part of corporations are small and medium-sized and require investment and sources for innovation (71). Likewise, empirical study on the relationship between PC and sustainable innovation ambidexterity is still inadequate and insufficient (31, 69, 70).

Moreover, the findings of this study also revealed a positive and significant relationship between STL and POS. The results are somewhat similar to a study by Suifan et al. (66) that examined the impact of transformational leadership on workers' innovation via the mediation impact of POS in the service industry of Jordan. The data was gathered from 369 workers employed in the Jordanian service industry. The hypotheses and mediation effects were analyzed utilizing SEM. The findings suggest that transformational leadership significantly impacts several aspects of workers' creativity and POS. Nevertheless, POS was not found in a significant association to several components of workers' creativity.

Finally, this study revealed direct mediation of PC on the relationships of STL on SIA and POS on SIA. Some other researchers, similarly used psychological capital as a mediator. One research study used PC as a mediator, between the relationship of transformational leadership and turnover intent. Corresponding to the conclusions, PC did not have any mediation impact on the aforementioned relationship (41). The conclusions of additional research underlined the mediation impact of PC in the association between transformational leadership and innovation ability. The conclusions emphasized the mediation impact of PC for the mentioned relationship (69). Finally, this research study also indicated a direct mediation of POS between the relationship of STL and PC. Similar research also studied the mediating effect of POS. The mediating impact of POS was analyzed on the correlation between transformational leadership and workers' inventiveness, however, the mediation was not found to be significant (66).

## Theoretical Implications

This research participates in several aspects of the earlier research on STL. Previous research has examined merely the part of transformational leadership on sustainable internal and external motivation (105), sustainable creativity (27), and sustainable efficiency (106). Hence, this research improves the intelligence flow of STL in a green building context by examining the link between STL and SIA. Alola et al. (107), The sustainability of any corporation is contingent on the involvement of workers. Therefore, the involvement of STL will lead the workers toward concern regarding the atmosphere and push the workers in the direction of attaining ecological and environmentally friendly objectives (24).

Moreover, this study improves the existing literature by analyzing workers' sustainable results via STL (108). This research suggests a substantially constructive influence between STL and PC of workers. The increasing consequences of a leader's efforts in encouraging workers' optimistic mindset in the direction of the company have been acknowledged by renowned investigators (25, 26) emphasizing that STL is essential to the company and the workers. This research contributes to the existing knowledge that employees with sustainable transformational leadership exhibit high PC, POS, and SIA (24).

## Managerial Implications

The discoveries of this research suggest numerous essential recommendations for green building industry managers. First, STL motivates encouraging adjustments, revitalizes, and its concerns regarding the employees, and promotes getting the employees organized for their achievement as a group. Likewise, consistent with the conclusions of earlier findings, which implied transformational leadership's significant impact on a group of employees 24, (109). Sustainable human resource development is an essential characteristic of sustainable human resource management, and in the service industry comprising a diversified workforce and customers (110). Consequently, green building supervisors ought to consider human resources knowledge and improvement. Through the redevelopment and growth of human resources, a sustainable competitive benefit can be attained (41). Therefore, employing the sustainability methodology in lowering the turnover intent of workers in the green building industry can be attained through constant STL education and improvement of managers' leadership capability and competencies. Leaders can further achieve prompt encouraging reactions, mindsets, and conduct in their workers. Additionally, the research backed the outcomes of previous studies demonstrating workers, that had positive experiences of STL possessed greater degrees of PC (70, 111).

The findings of this research similarly offer valuable understandings for recognizing the elements of PC completely and sustainable innovation ambidexterity in the green building industry. Supervisors are recommended to improve their workers' abilities and PC by continuous evaluation along with utilizing development strategies with the aim to offer greater efficiency. Additionally, owing to the crucial part of innovation, supervisors are suggested to encourage innovative sustainable concepts to acquire competitive lead via utilizing and supporting innovative atmosphere especially in service provision and contemplating clients' demands. Additionally, regarding the components of PC, supervisors can improve every one of the components by designing and strengthening a valuable atmosphere with the purpose to improve the PC and sustainable innovation in their companies (31). The mixture of all PC elements implies this point to the legislators to consider all aspects altogether and not one at a time. Consequently, a methodical approach is chosen to improve the workers' PC in green building companies.

## Limitations and Future Research Directions

This study offered substantial potential investigation prospects. It is crucial to discover the efficacy of motivations in more countries, provided that the conclusions of the usefulness of motivations in this study are mostly tilted to China. In Europe and various areas of South Africa, and Asia, green building methods have been effectively established, and the application of encouragements to support green building procedures is highly developed. It will be helpful to understand the efficiency of motivations as tools for advocating green building practices. This will likewise advise the universality of the usefulness of motivations as methods for handling green building practices. Additionally, as motivations, particularly government motivations, sustain a massive investment. It is essential to evaluate their efficacy with the aim to rationalize the ongoing investment. Moreover, this investigation employed a cross-sectional study, consequently, potential researchers should contemplate applying a longitudinal layout with data attained from a distinct resource to estimate fundamental associations between the constructs further precisely. This will additionally promote the awareness of the investigation, particularly in the green building industry.

This research only examines the impact of STL on workers' PC and SIA centered on the attributes of transformational leaders in common. A potential investigation should analyze enhance the association between particular methods of STL and these concepts to get a brighter path important to innovation in the situation of a developing country context. Finally, STL is considered as the factors of both innovation and various managerial results for instance corporate citizenship conducts, transformation management, education management, worker satisfaction, and loyalty (71), potential works must examine the consequences of STL on the additional tactical constructs to take advantage of STL's ability and advantages. The existing study also offers additional useful understandings for potential investigations, that should analyze the performance consequences of innovation and the constructs that can moderate the association between PC and SIA. It is additionally proposed that potential research might require putting up works on the relative investigations to discover and assess thoroughly the constructs that could impact SIA in green building industries.

## Data Availability Statement

The original contributions presented in the study are included in the article/supplementary material, further inquiries can be directed to the corresponding author/s.

## Ethics Statement

Ethical review and approval were not required for the study on human participants in accordance with the local legislation and institutional requirements. Informed consent was obtained from all subjects involved in the study.

## Author Contributions

YT conceived and designed the research, wrote, and revised the manuscript. Y-JC, Y-FS, and QC gave guidance throughout the whole research process. All authors contributed to the article and approved the submitted version.

## Funding

This research was supported by Sichuan Science and Technology Program (Grant Numbers: 2020JDR0239 and 2021JDR0150), Research Center for System Science and Enterprise Development (Grant Number: Xq21B08), Cooperation Project of Ministry of Education of China (Grant Number: 202101364053), Sichuan Wine Development Research Center (Grant Number: CJZB20-02), Planning Office of Sichuan Federation of Social Sciences Associations (Grant Number: SC19EZD049), and National Science Foundation of China (Grant Numbers: 72172024 and 71872027).

## Conflict of Interest

The authors declare that the research was conducted in the absence of any commercial or financial relationships that could be construed as a potential conflict of interest.

## Publisher's Note

All claims expressed in this article are solely those of the authors and do not necessarily represent those of their affiliated organizations, or those of the publisher, the editors and the reviewers. Any product that may be evaluated in this article, or claim that may be made by its manufacturer, is not guaranteed or endorsed by the publisher.
